# Association between the planetary health diet index and risk of cardiovascular diseases in postmenopausal women: Findings from the Women’s Health Initiative Observational Study

**DOI:** 10.1016/j.ajpc.2026.101750

**Published:** 2026-07-17

**Authors:** Donya Shahamati, Bo Yang, Simin Liu, Andrew O. Odegaard, Matthew Allison, Andrea J. Glenn, Su Yon Jung, Matthew Nudy, Peter F. Schnatz, Aladdin H. Shadyab, James M. Shikany, Lesley F. Tinker, Robert B. Wallace, Yujue Wang, Matthew J. Landry

**Affiliations:** a Department of Global Health & Biobehavioral Sciences, Joe C. Wen School of Population & Public Health, University of California, Irvine, CA, USA; b Department of Epidemiology and Biostatistics, Joe C. Wen School of Population & Public Health, University of California, Irvine, CA, USA; c Center for Global Cardiometabolic Health and Nutrition, University of California, Irvine, CA, USA; d Mary & Steve Wen Cardiovascular Division, Department of Medicine, School of Medicine, University of California, Irvine, CA, USA; e Department of Family Medicine, School of Medicine, University of California, San Diego, La Jolla, CA, USA; f Department of Nutrition and Food Studies, Steinhardt School of Culture, Education, and Human Development, New York University, NY, USA; g Department of Nutrition, Harvard T.H. Chan School of Public Health, Boston, MA, USA; h Translational Sciences Section, Department of Epidemiology, Jonsson Comprehensive Cancer Center, School of Nursing, Fielding School of Public Health, University of California, Los Angeles, CA, USA; i Department of Medicine and Public Health Sciences, Penn State College of Medicine, The Pennsylvania State University, Hershey, PA, USA; j Department of Obstetrics and Gynecology & Internal Medicine, Rowan-Virtua School of Osteopathic Medicine, Stratford, NJ, USA; k Herbert Wertheim School of Public Health and Human Longevity Science and Division of Geriatrics, Gerontology, and Palliative Care, Department of Medicine, University of California, San Diego, La Jolla, CA, USA; l Division of General Internal Medicine and Population Science, Department of Medicine Heersink, School of Medicine, University of Alabama at Birmingham, Birmingham, AL, USA; m Cancer Prevention Program, Public Health Sciences Division, Fred Hutchinson Cancer Center, Seattle, WA, USA; n Department of Epidemiology and Internal Medicine, College of Public Health, University of Iowa, Iowa City, IA, USA

## 1 Introduction

During the menopausal transition, several cardiometabolic factors may become less favorable, including increased central adiposity, adverse changes in lipids and insulin sensitivity, higher blood pressure and impaired vascular function. These changes may contribute to higher cardiovascular risk during the postmenopausal years [1,2]. Because diet is modifiable at both individual and population levels, and is closely related to cardiometabolic health, dietary behavior is an important part of cardiovascular disease (CVD) prevention [3].

The Planetary Health Diet (PHD), introduced by the EAT-Lancet Commission, connects human health and environmental sustainability. It emphasizes higher intake of diverse plant-based foods, lower intake of animal-source foods, preference for unsaturated fats over saturated fats, and limited intake of refined grains and added sugars [4]. The Planetary Health Diet Index (PHDI) was developed to quantitatively assess alignment with the PHD. In this index, dietary intake is scored based on how closely consumption of key food groups aligns with the EAT-Lancet recommended intake ranges [5]. Prior longitudinal cohort studies have reported that higher PHDI scores are associated with lower risk of incident CVD, including coronary heart disease (CHD), and stroke [6,7]. We examined the association between baseline PHDI scores and incident total CVD, CHD, stroke, and heart failure in the Women’s Health Initiative Observational Study (WHI-OS).

## 2 Methods

We analyzed data from the WHI-OS, a prospective cohort of postmenopausal women in the United States. Women were eligible for this analysis if they had complete baseline dietary data and follow-up information through February 2025. We excluded participants with known CVD at baseline, implausible total energy intake (<600 or >5000 kcal/day), missing dietary or key covariate data, missing follow-up time, or loss to follow-up after baseline. The final analytic sample included 66,892 women. All participants provided written informed consent, and WHI protocols were approved by institutional review boards at participating institutions.

We used the baseline PHDI score as the main exposure. This score was calculated from dietary intake reported on the WHI food frequency questionnaire (FFQ) and was used to represent each participant’s alignment with the EAT-Lancet dietary recommendations. After harmonizing WHI dietary variables into the units required for PHDI scoring, component scores were summed to create a total score ranging from 0 to 140, with higher scores indicating greater alignment. We examined PHDI as both quintiles and as a continuous score. For the main Cox proportional hazards regression models, PHDI was modeled continuously per 10-point higher score. We also evaluated Healthy Eating Index-2015 (HEI-2015) and Alternative Healthy Eating Index-2010 (AHEI-2010) to compare PHDI with established diet-quality indices.

The primary outcome was incident total CVD, defined as the first occurrence of CHD, stroke, or heart failure. Secondary outcomes included CHD, stroke, ischemic stroke, hemorrhagic stroke, and heart failure. Outcomes were physician-adjudicated. Participants were censored at death, loss to follow-up, or end of follow-up, whichever occurred first.

Baseline characteristics and PHDI component scores were described across PHDI quintiles. We also calculated the number of events, person-years, and crude incidence rates per 1000 person-years for total CVD and each individual CVD outcome. To examine the association between PHDI and CVD outcomes, Cox proportional hazards models were used, and results were reported as hazard ratios (HRs) and 95% confidence intervals (CIs). The adjusted models included age, race/ethnicity, education, smoking, alcohol intake, physical activity, total energy intake, and body mass index (BMI). To better understand whether the PHDI findings were independent of overall diet quality, Pearson correlations were calculated between PHDI, HEI-2015, and AHEI-2010. Additional models were then run with further adjustment for HEI-2015 or AHEI-2010. Another sensitivity analysis additionally adjusted for baseline hypertension, diabetes, and self-reported high cholesterol requiring medication. We also conducted a Fine–Gray competing-risk analysis, treating non-CVD death as a competing event, and exploratory subgroup analyses by age, race/ethnicity, BMI category, diabetes status, hypertension and smoking status. Analyses were conducted using a complete-case approach and were performed in R.

## 3 Results

The analytic sample included 66,892 postmenopausal women. Baseline PHDI scores ranged from approximately 35 to 123 (mean ± SD; 75.5 ± 10.3). Mean PHDI scores increased across quintiles, from approximately 61 in Q1 to 90 in Q5. Selected baseline characteristics across PHDI quintiles are shown in Fig. 1A. Compared with women in Q1, those in Q5 were slightly older, had higher HEI-2015 and AHEI-2010 scores, lower BMI, and higher levels of recreational physical activity.

**Fig. 1:**
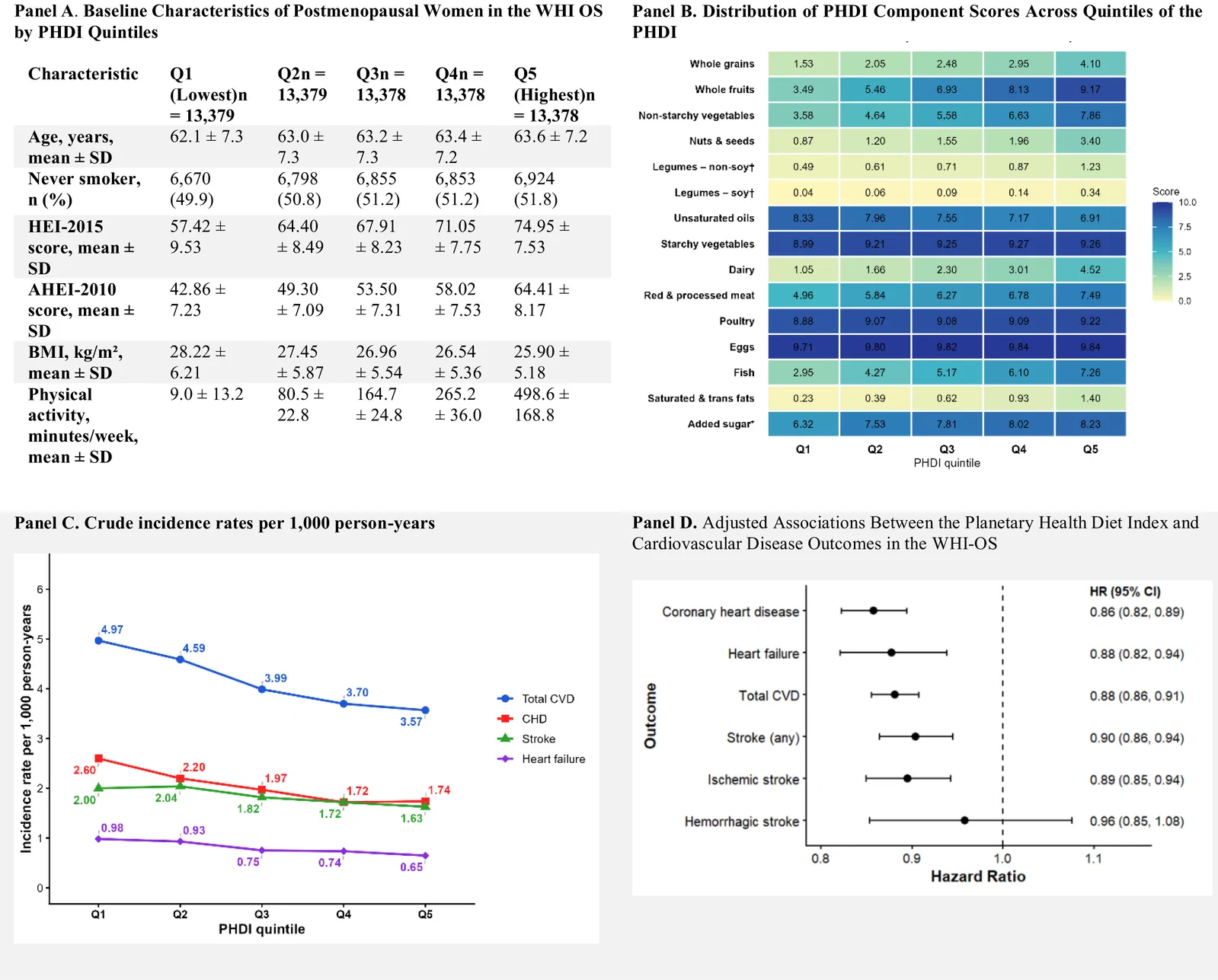
Panel A. Selected baseline characteristics across PHDI quintiles. Values are expressed as mean ± standard deviation. Physical activity was assessed as minutes of recreational physical activity per week, including walking and mild, moderate, and strenuous physical activity. Panel B. Distribution of PHDI component scores across quintiles of total PHDI score. Non-soy legumes and soy foods together contribute a maximum of 10 points to the total PHDI score. Added sugar was used for the moderation component in place of combined added sugar and fruit juice due to unit harmonization constraints. Panel C. Crude incidence rates of cardiovascular outcomes across PHDI quintiles, expressed per 1000 person-years. Panel D. Adjusted associations between PHDI and cardiovascular outcomes per 10-point higher PHDI score. Hazard ratios and 95% confidence intervals were estimated using Cox proportional hazards models; the dashed vertical line indicates the null value. Abbreviations: AHEI-2010, Alternative Healthy Eating Index-2010; BMI, body mass index; CHD, coronary heart disease; CI, confidence interval; CVD, cardiovascular disease; HEI-2015, Healthy Eating Index-2015; HR, hazard ratio; PHDI, Planetary Health Diet Index; WHI-OS, Women’s Health Initiative Observational Study.

Component-level scoring patterns are shown in Fig. 1B. Higher total PHDI scores corresponded with higher component scores for plant-forward food groups, including whole grains, whole fruits, non-starchy vegetables, nuts and seeds, legumes, and fish. However, legumes, particularly soy foods, nuts and seeds remained among the lowest-scoring plant-based components across quintiles. Saturated and trans fats also had low scores across quintiles.

Over a median follow-up of 19.6 years (IQR: 8.9–26.8), 5033 incident total CVD events occurred. Crude incidence rates decreased across PHDI quintiles for most outcomes (Fig. 1C). After adjustment for covariates, higher PHDI scores were associated with lower hazards of most CVD outcomes, with a decreasing pattern across PHDI quintiles (Fig. 1D). For total CVD, lower hazards were evident from Q2 through Q5 compared with Q1 (Q2 HR: 0.86; 95% CI: 0.78–0.95; Q5 HR: 0.72; 95% CI: 0.64–0.81; p-trend <0.001). Similar patterns were observed for CHD, stroke, ischemic stroke, and heart failure, but not hemorrhagic stroke. Moreover, for each 10-point higher PHDI score, the hazard was lower for total CVD, CHD, stroke, ischemic stroke, and heart failure. The association with hemorrhagic stroke was not statistically significant.

PHDI was moderately correlated with HEI-2015 (r = 0.607) and more strongly correlated with AHEI-2010 (r = 0.734). The association was attenuated after additional adjustment for HEI-2015 (Q5 vs Q1 HR: 0.80; 95% CI: 0.71–0.90; p-trend <0.001) and further attenuated after AHEI-2010 adjustment (Q5 vs Q1 HR: 0.89; 95% CI: 0.78–1.02; p-trend = 0.034). Separately, after additional adjustment of the primary model for baseline hypertension, diabetes, and self-reported high cholesterol requiring medication, the association with total CVD remained inverse (Q5 vs Q1 HR: 0.70; 95% CI: 0.64–0.77; p-trend <0.001); inverse associations remained significant for CHD, stroke, ischemic stroke, and heart failure, but not for hemorrhagic stroke. In a Fine–Gray competing-risk analysis treating non-CVD death as a competing event, the association between higher PHDI and total CVD remained inverse (Q5 vs Q1 HRs: 0.78; 95% CI: 0.71–0.86). Exploratory subgroup analyses showed generally consistent inverse associations across age, race/ethnicity, BMI, diabetes, hypertension, and smoking groups, with no statistically significant interactions.

## 4 Discussion

In this large prospective cohort of postmenopausal women from the WHI-OS, women in the highest PHDI quintile had a 28% lower hazard of total CVD compared with those in the lowest quintile over long-term follow-up. These associations were also observed for CHD, stroke, ischemic stroke, and heart failure, but not for hemorrhagic stroke.

Our findings are generally consistent with prior longitudinal cohort studies reporting inverse associations between PHDI scores and CVD outcomes in other populations [6,7]. The null association observed for hemorrhagic stroke may reflect differences in underlying mechanisms and the smaller number of hemorrhagic stroke events.

The association between PHDI and CVD may overlap with general diet quality, especially because PHDI was correlated with HEI-2015 and more strongly with AHEI-2010. However, PHDI is different from these indices because it is based on the PHD, which was developed to consider both human health and environmental sustainability [4,5]. In this sense, our findings may be useful when considering dietary patterns that support cardiovascular health and may also be compatible with more sustainable food systems. However, we did not measure environmental or food-system outcomes in this study. Therefore, this interpretation should be viewed as broader context rather than a direct finding from our analysis.

Several limitations should be considered. Diet was assessed using a baseline FFQ, which may introduce measurement error and does not capture dietary changes or adherence over time. Although models adjusted for sociodemographic and lifestyle factors, women with higher PHDI scores had more favorable health-related characteristics. Therefore, healthy-user bias and residual confounding cannot be excluded, and the findings should be interpreted as observational associations rather than causal effects. Results were robust in competing-risk analysis and generally consistent across exploratory subgroups. The analyses in this report provide evidence that greater alignment with the PHD is associated with lower hazards of multiple CVD outcomes among postmenopausal women. By extending prior findings to the WHI, these results underscore the relevance of plant-forward dietary patterns in later life and support further investigation into how dietary quality and sustainability-oriented eating patterns intersect with cardiovascular health in aging populations.

## Data availability

The Women’s Health Initiative (WHI) data are available to qualified researchers. Deidentified individual participant data, along with study protocols, procedures, and data collection forms, are accessible through the WHI online resource (https://www.whi.org/datasets) and through the National Heart, Lung, and Blood Institute’s BioLINCC repository (https://biolincc.nhlbi.nih.gov/studies/whi_ctos/). Access is granted to eligible researchers with an approved research proposal in accordance with WHI and NHLBI guidelines.

## Funding

The WHI program is funded by the 10.13039/100000050National Heart, Lung, and Blood Institute, 10.13039/100000002National Institutes of Health, 10.13039/100000016U.S. Department of Health and Human Services through contracts 75N92021D00001, 75N92021D00002, 75N92021D00003, 75N92021D00004, 75N92021D00005. The content is solely the responsibility of the authors and does not necessarily represent the official views of the NIH.

## Declaration of generative AI and AI-assisted technologies in the writing process

The author(s) declare that no generative AI or AI-assisted technologies were used in the writing of this manuscript.

## CRediT authorship contribution statement

**Donya Shahamati:** Writing – review & editing, Writing – original draft, Visualization, Methodology, Formal analysis, Conceptualization. **Bo Yang:** Writing – review & editing, Methodology, Formal analysis. **Simin Liu:** Writing – review & editing, Conceptualization. **Andrew O. Odegaard:** Writing – review & editing, Conceptualization. **Matthew Allison:** Writing – review & editing. **Andrea J. Glenn:** Writing – review & editing. **Su Yon Jung:** Writing – review & editing. **Matthew Nudy:** Writing – review & editing. **Peter F. Schnatz:** Writing – review & editing. **Aladdin H. Shadyab:** Writing – review & editing. **James M. Shikany:** Writing – review & editing. **Lesley F. Tinker:** Writing – review & editing. **Robert B. Wallace:** Writing – review & editing. **Yujue Wang:** Writing – review & editing. **Matthew J. Landry:** Writing – review & editing, Writing – original draft, Visualization, Supervision, Methodology, Formal analysis, Conceptualization.

## Declaration of competing interest

The authors declare that they have no known competing financial interests or personal relationships that could have appeared to influence the work reported in this paper.
